# Students with global experiences during medical school are more likely to work in settings that focus on the underserved: an observational study from a public U.S. institution

**DOI:** 10.1186/s12909-021-02975-3

**Published:** 2021-10-29

**Authors:** Shay E. Slifko, Nadja A. Vielot, Sylvia Becker-Dreps, Donald E. Pathman, Justin G. Myers, Martha Carlough

**Affiliations:** 1 Office of Global Health Education, Institute for Global Health & Infectious Diseases, University of North Carolina at Chapel Hill, 1002 Bondurant, CB# 9535, Chapel Hill, USA; 2 Department of Family Medicine, University of North Carolina, Chapel Hill, USA; 3 Department of Epidemiology, University of North Carolina Gillings School of Global Public Health, Chapel Hill, USA; 4 Department of Emergency Medicine, University of North Carolina, Chapel Hill, USA; 5 Department of Maternal and Child Health, University of North Carolina Gillings School of Global Public Health, Chapel Hill, USA

**Keywords:** Medical students, Global Health, Global Health electives, Underserved, medical education, Career choice

## Abstract

**Background:**

Global health interest has grown among medical students over the past 20 years, and most medical schools offer global health opportunities. Studies suggest that completing global health electives during medical school may increase the likelihood of working with underserved populations in a clinical or research capacity. This study aimed to assess the association of global electives in medical school on subsequently working in global health and with underserved populations in the United States (U.S.), additionally considering students’ interests and experiences prior to medical school. We also examined whether respondents perceived benefits gained from global electives.

**Methods:**

We surveyed medical school graduates (classes of 2011-2015) from a large public medical school in the U.S. to describe current practice settings and previous global health experience. We evaluated work, volunteer, and educational experiences preceding medical school, socioeconomic status, race and ethnicity using American Medical College Application Service (AMCAS) data. We assessed the association between students’ backgrounds, completing global health electives in medical school and current work in global health or with underserved populations in the U.S.

**Results:**

In the 5 to 8 years post-graduation, 78% of 161 respondents reported work, research, or teaching with a focus on global or underserved U.S. populations. Completing a global health elective during medical school (*p* = 0.0002) or during residency (*p* = 0.06) were positively associated with currently working with underserved populations in the U.S. and pre-medical school experiences were marginally associated (*p* = 0.1). Adjusting for pre-medical school experiences, completing a global health elective during medical school was associated with a 22% greater prevalence of working with an underserved population. Perceived benefits from global electives included improved cultural awareness, language skills, public health and research skills, and ability to practice in technology-limited settings.

**Conclusion:**

Medical school graduates who participated in global electives as students were more likely than their peers to pursue careers with underserved populations, independent of experiences prior to medical school. We hypothesize that by offering global health experiences, medical schools can enhance the interests and skills of graduates that will make them more likely and better prepared to work with underserved populations in the U.S. and abroad.

**Supplementary Information:**

The online version contains supplementary material available at 10.1186/s12909-021-02975-3.

## Background

Medical school experiences can play a formative role in student career decision making and can have substantial influence on future practice choices, both in terms of medical specialties pursued and populations served. These experiences may be realized through formal curricular components, clinical training sites, and elective opportunities, all of which can have a focus on underserved patient populations. Many underserved populations in the U.S. lack high-quality and accessible primary health care, increasing the likelihood of experiencing poor health and reduced life expectancy. Increasing the work force of primary care physicians in underserved areas is needed to meet the medical needs of populations living in these areas (Mullen, F. et al., 2010).

Opportunities for medical students to learn skills important for working with underserved populations include clinical training in rural and urban settings; language immersion programs; migrant farmworker outreach; refugee health initiatives; and short-term global health experiences in low- and middle- income countries [10]. Medical students and resident physicians develop stronger preferences for serving underserved populations as compared to their peers when they have engaged in global health experiences [3, 5, 7]. Furthermore, students who attended a medical school with a strong social mission or grew up in a medically underserved setting are also more likely to work with the underserved [11].

In low- and middle- income countries, patient populations face challenges like those of underserved populations in the U.S.: healthcare provider shortages, limited access to adequate healthcare services, low health literacy, long travel distances, and high financial burdens for families to obtain health care [7]. Global health electives during medical school may provide students the opportunity to learn from resourceful healthcare workers in often constrained systems; develop a deeper understanding of the barriers to healthcare, learn about public health systems in other countries, and contribute to research studies to address a high priority health problem in the population. Global health engagement has increased rapidly within both medical school and residency programs and the reported benefits include improved medical knowledge, procedural skills, increased recognition of cost-effective practices and improvement in cultural literacy [6, 9]. Graduates report a greater understanding of the social determinants of health, intentions to practice in rural settings, and commitment to care for the medically underserved [8]. Yet, more information is needed to understand how global medical education programs can help build the numbers of medical students who subsequently pursue work in underserved communities, both within and outside the U.S [Puddey, IB et al., 2015).

To this end, the study team surveyed graduates of a large public medical school to understand whether participating in global health electives as medical students is associated with practice settings with underserved populations. We also evaluated the influence of other factors, including sociodemographic factors and experiences in global health and underserved populations prior to entering medical school. Finally, we summarized participants’ perceptions of their global health experiences and the perceived positive and negative effects for their careers. These findings may be used by medical schools interested in adapting their curricula to assess whether global health experiences produce graduates more likely and better equipped to serve vulnerable populations.

## Methods

### Study population and recruitment

Eligible study participants were 2011 through 2015 graduates from a large public medical school in a medium-sized city setting within the southeastern United States. The school had a traditional 2 + 2 curriculum during the 2011-2015 school years, with an option for an elective but no required global health experience. The study team identified eligible alumni and email addresses from an opt-in alumni registry maintained by the school, and emailed study invitations to all eligible alumni with periodic email reminders to those who had not yet responded. A $20 electronic café gift card was offered for participation.

### Data sources and study measures

The study team specifically designed and generated a self-administered, web-based survey using Qualtrics© software for this study (see Additional File; Qualtrics© Software version 2019). The study questionnaire was created using a modified delphi process by faculty and staff within the Office of Global Health Education who are active in global health and medical student education. Building on prior research [1], a retrospective survey about global health learning experiences was piloted among medical students and faculty interested and active in global health. Global health electives at this medical school institution have developed considerably over the last decade and structured opportunities in global health research, community health experiences, and clinical rotations at a minimum of 4-8 weeks are available. Each of the electives require advance planning, faculty mentorship, and approval and evaluation by an on-site preceptor. Many of them include the preparation of scholarly projects, including completion of short reports, essays and papers intended for publication.

In addition to demographic characteristics, the survey solicited information on participants’ current employment situation, location, and characteristics of their current patient populations. The principal exposure of interest was whether participants reported completing a global elective during medical school. Participants were also asked about details of global elective experiences during medical school, completion of global health experiences during residency, motivations for participating in the experience, perceived benefits of the experience, skills gained from the experience, and subsequent completion of global health experiences during residency.

We defined the principal outcome of interest as whether participants reported working with underserved populations in their current work settings, including:

1. Clinical practice with a population that is typically medically underserved, including in federal or state health professional shortage areas; Indian, tribal, or migrant health facilities; community health centers (including mental health); rural health centers; critical access hospitals; correctional facilities.
2. Clinical practice (e.g., with immigrant populations), research, teaching, or programmatic work with a global focus from the U.S.; or
3. Clinical practice in a global setting.

The survey opened May 7, 2019, and closed July 24, 2019. To augment survey data, the study team obtained permission from our school of medicine’s administrators to access data that students provided in their American Medical College Application Services (AMCAS) applications when they had applied to medical schools (https://students-residents.aamc.org/media/5186/download). The pre-medical school entry data consisted of demographics and information on work and volunteer experiences prior to applying to medical school, as well as major and minor areas of undergraduate study. From the AMCAS data, work and volunteer experiences prior to medical school were considered global in nature if they included a study abroad program; study or use of a foreign (i.e., not English) language; research, clinical work, or volunteerism in a global setting; or a medical mission. Experience was focused on a special population if it included interaction or research in individuals who were impoverished or homeless, victims of violence, or people living with HIV/AIDS. Undergraduate majors and minors were global in nature if they included study of a foreign language or global relations.

### Data analysis

We summarized participant characteristics with univariate frequencies and percentages. We used the Mantel-Haenszel chi-squared test to identify differences in the proportions of participants who experienced the outcome by participation in global electives and other variables of interest, including demographic characteristics and work and volunteer experience prior to medical school.

After assessing bivariate associations, we ran multivariable log-binomial models to estimate the relative prevalence of the outcome by whether participants completed a global elective during medical school. Additional variables that were associated with the outcome at *p* ≤ 0.1 in bivariate analyses were assessed as potential confounders using a causal diagram, and these variables were retained in the final parsimonious model.

Finally, the study team summarized participants’ perceptions of how global elective experiences affected their careers using descriptive statistics and illustrative quotations. Responses suggested the elective provided useful patient-care skills, made the participant more competitive for future career opportunities, or served as a morally edifying experience were considered positive. Responses suggesting that the elective did not have an impact on one’s career path, or has not yet had an impact, were considered neutral. Responses suggesting that the elective served as an impediment to skills development or career advancement were considered negative. Following these guidelines, qualitative responses were independently reviewed and categorized as positive, negative, or neutral by two authors (SES, NAV). Discrepancies between authors were resolved by joint discussion and consultation with a third party, when necessary.

Missing data for exposure and outcome variables were excluded from the analysis without imputation. All analyses were completed using SAS version 9.4 (Research Triangle Park, North Carolina). A *p*-value level of statistical significance was set at = 0.05. The study was approved by the Institutional Review Board of the University of North Carolina at Chapel Hill (#18-2374).

## Results

### Characteristics of the sample

The study team identified 824 alumni who graduated between 2011 and 2015. Thirty-two email addresses proved invalid, resulting in 792 presumed contacts with alumni. Of these, 172 alumni (21.7%) responded to the survey, of whom 161 provided data on the outcome and were included in analyses; three participants who reported being unemployed were excluded. Based on data from AMCAS applications, most of the 161 eligible participants identified as female (61.5%), identified as White (76.3%) and non-Latinx (96.3%) and were ages 30-34 years at the time of the survey (57.1%) (Table 1). Nearly all participants (99.3%) spoke English as their primary language, and 7.5% reported growing up socioeconomically disadvantaged.

**Table 1:** Characteristics of respondents of medical student classes of 2011-2015 (*n* = 161)

Characteristics	***N*** (%)
***Demographics***	
Female sex	99 (61.5)
Race *(2 missing)*	
African American	9 (5.6)
Asian	16 (10.01)
Native American	1 (0.6)
White	122 (76.3)
More than one race	1 (0.6)
Not reported/Other	12 (7.5)
Ethnicity *(1 missing)*	
Not Latinx	160 (96.3)
Mexican American/Chicanx/Puerto Rican/Spanish/Latinx	6 (3.7)
Age group (years)	
25-29	3 (1.9)
30-34	92 (57.1)
35-39	55 (34.2)
40+	11 (6.8)
Disadvantaged economically by self-report *(13 missing)*	12 (7.5)
Applicant’s native language is English *(13 missing)*	147 (99.3)
***Experience prior to medical school***	
College major/minor global in nature^a^	21 (13.0)
Pre-med school work or volunteer experience global in nature^b^ *(13 missing)*	80 (54.1)
Pre-med school work or volunteer experience focused on special populations^c^ *(13 missing)*	34 (23.0)
***Global health experiences during and after medical school***	
Completed a global elective in medical school	76 (47.2)
Completed a global elective in residency *(6 missing)*	50 (32.3)
Medical student loan debt ≥ $100,00 *(7 missing)*	82 (53.2)
***Current medical activities***	
Current position^d^	
Resident	20 (12.4)
Fellow	35 (21.7)
Outpatient provider	77 (47.8)
Inpatient provider	48 (29.8)
Administrator	12 (7.5)
Other	13 (8.1)
Currently practicing outside of the United States *(1 missing)*	4 (2.5)
Working in non-metropolitan statistical area < 50,000 population *(16 missing)*	153 (95.0)
Currently working in a practice serving special communities^d^	
No	50 (31.1)
Health professional shortage area	10 (6.2)
State designated shortage area	2 (1.2)
Federally designated health center	21 (13.0)
Not federally designated health center	95 (59.0)

^a^Includes foreign language and international relations majors and minors

^b^Includes study abroad, language-based, globally-focused research or clinical work, and medical missions

^c^Includes work with impoverished or homeless populations, people living with HIV/AIDS, and victims of violence

^d^Total may sum to more than 100% as multiple responses were possible

Participants were a median 6 years post-graduation at the time of the survey (interquartile range: 5-7 years). Fifty-five of the 161 participants were still in training, including 20 residents and 35 fellows (Table 1). Most participants (54.1%) reported performing work or volunteer experiences with a global focus and one-quarter (23.0%) reported an experience working with a special population prior to attending medical school (Table 1). Seventy-six participants (47.2%) reported completing a global elective during medical school, 37 of whom also reported completing a global elective during residency.

### Correlates of working with underserved populations

Of 161 participants who provided data on the primary outcome (“working with underserved populations”), 125 (77.6%) reported practicing with a medically underserved population in the U.S. (*n* = 31); clinical practice, research, teaching, or programmatic work with a global focus from the U.S. (*n* = 121, including 111 who reported clinical practice with immigrant populations in the U.S.); and practicing in a global setting (*n* = 4). The percentage of participants who reported working with underserved populations was higher among those who had completed a work or volunteer experience with a global focus prior to medical school (82.5% vs. 72.1%, *p* = 0.1), or completed a global elective in medical school (90.8% vs. 65.9%, *p* = 0.0002) or residency (91.9% vs. 76.9%, *p* = 0.06) (Table 2). Participant sex, race/ethnicity, and burden of student loan debt were comparable between participants who did and did not work with underserved populations.

**Table 2:** Predictors of working in a global setting, in globally-focused activities, or with special populations among medical school classes of 2011-2015 (*n* = 161)

	Works with underserved populations n (%)	Does not work with underserved populations n (%)	***p***
Sex			
Female (*n* = 99)	80 (80.8)	19 (19.2)	0.2
Male (*n* = 62)	45 (72.6)	17 (27.4)	
Race/Ethnicity *(11 missing)*			
White, non-Latinx (*n* = 122)	95 (77.7)	27 (22.1)	0.9
Non-white, including Latinx (*n* = 39)	30 (76.9)	9 (23.1)	
Amount of medical student loan debt *(7 missing)*			
≥ $100,000 (*n* = 82)	64 (78.1)	18 (21.9)	0.8
< $100,000 (*n* = 72)	55 (76.4)	17 (23.6)	
College major/minor had global focus			
Yes (*n* = 21)	19 (90.5)	2 (9.5)	0.2
No (*n* = 40)	106 (75.7)	34 (24.3)	
Completed work/volunteer experience with global focus prior to medical school *(13 missing)*			
Yes (*n* = 80)	66 (82.5)	14 (17.5)	0.1
No (*n* = 68)	49 (72.1)	19 (27.9)	
Completed work/volunteer experience with special populations in the U.S. prior to medical school *(13 missing)*			
Yes (*n* = 34)	29 (85.3)	5 (14.7)	0.2
No (*n* = 114)	86 (75.4)	28 (24.6)	
Completed a global elective in medical school			
Yes (*n* = 76)	69 (90.8)	7 (9.2)	0.0002
No (*n* = 85)	56 (65.9)	29 (34.1)	
Completed a global elective in residency among non-trainees *(n = 102, 3 missing)*			
Yes (*n* = 37)	34 (91.9)	3 (8.1)	0.06
No (*n* = 65)	50 (76.9)	15 (23.1)	

*p-values for variables with cell sizes < 5 were estimated using Fisher’s Exact Test

We identified experiences prior to medical school as a potential confounder, and experiences during residency as an intermediary factor, in the association between medical school experiences and working with underserved populations. Because not all respondents had completed training at the time of the survey, and thus might not have had an opportunity to complete a global health experience as a trainee, we developed two models. The first model included all respondents, regardless of their stage in training, and adjusted for experiences prior to medical school to estimate the direct association of medical school experiences on working with underserved populations. The second model restricted to respondents who had completed residency and fellowship, further adjusted for experiences during to estimate the direct association of medical school experiences. In both models we identified a 22-25% increased prevalence of working with underserved populations among participants who completed a global health elective in medical school, adjusting for experiences prior to medical school and experiences during residency (where applicable) (Table 3).

**Table 3:** Multivariable correlates of working with underserved populations among participants from medical school classes 2011-2015 (*n* = 161)

Covariate	Adjusted Prevalence Ratio^a^ (95% Confidence Interval)	
	Model 1: All respondents (*n* = 161)	Model 2: Restricted to respondents who had completed training (*n* = 106)
Completed a global elective in medical school	1.22 (1.01, 1.48)	1.25 (1.02, 1.54)
Work/volunteer experience with global focus before medical school	1.08 (0.94, 1.25)	1.10 (0.94, 1.28)
Completed a global elective in residency	Not estimated	1.10 (0.89, 1.34)

^a^Each prevalence ratio is adjusted for all other estimated covariates in the table

### Perceived benefits of global health electives

Most participants reported that the global experience improved their cultural competency (72.4%) and understanding of public health topics and methods (71.1%) (Table 4). Twelve percent reported that the experiences improved their research skills, while a substantial percentage reported it improved their language skills, as well as improved clinical and diagnostic skills in low-technology settings (Table 4). When asked to describe how the experience influenced their ability to get the job they wanted (*n* = 53), 29 participants (54.7%) reported that the experience was a positive influence on their career paths:

*“My time in Central America gave me the opportunity to become proficient in medical Spanish. I used this skill every day during residency. My current job as an academic attending does not involve working with Hispanic immigrants. The lack of immigrants within my practice is the biggest reason that I am motivated to change jobs in the next 5 years. I miss it and didn't realize how important it was to my practice until I didn't have it anymore.” (Respondent spent 8 weeks in clinical, Spanish-speaking setting abroad as a student and reported spending up to a quarter of their annual work activities working with immigrants in the U.S.).*

“*Due to my immersion experience in Guatemala, I was able to see Spanish speaking patients throughout residency and now in my career I speak Spanish daily. It has made me a marketable provider to be bilingual and I have cultural competency regarding patients from Central America that I would not have had with simple language training in the U.S*.” (Participant reported practicing in a federal health professional shortage area, outpatient setting with immigrant and Medicaid populations in the U.S).

No participant identified the experience as negative, although 20 participants (37.7%) reported the experience had no influence on being hired in their desired job:

*“My experience during medical school impacted decisions I have made about my career subsequently, though I do not believe that it impacted my ability to get the job I wanted.”* (Respondent reported practicing in an academic setting with up to a quarter of their work activities per year dedicated to clinical activities conducted internationally, global health research, and working with immigrant populations in the U.S.).

*“It was certainly a talking point for job interviews, but I'm not sure that it really helped me get my current job in private practice. I hoped it would help me get an academic position where I could dedicate a significant portion of my time to global health work/education, however there are still not many places interested in hiring someone for that niche, as it doesn't generate a lot of revenue.”* (Respondent reported practicing in both inpatient and outpatient settings with up to a quarter of annual clinical activities conducted internationally).

Four participants (7.5%) indicated the experience seemed to have strengthened their candidacy for subsequent positions:

*“I think it probably helped with my applications to residency and fellowship. I am hoping to continue global health research for my fellowship research, so my med school global health experience was important when applying for fellowships and I think it probably helped my competitiveness as a candidate.”* (Participant reported practicing as a current Fellow and estimated up to 25% of their yearly work activities involved global health research conducted from the U.S. and working with immigrant populations in the U.S.).

*“These experiences provided further training in caring for underserved populations and informed my care of immigrant populations. The experiences were good to include on my CV [and] improved physician exam skills and diagnostic skills without technology”* (Participant reported practicing in outpatient settings with immigrant and Medicaid populations in the U.S.)

**Table 4:** Self-reported benefits of international electives among respondents of medical school classes of 2011-2015 (*n* = 78)

Skill	Substantial or profound influence n (%)
Improved clinical skills *(3 missing)*	24 (32.0)
Improved diagnostic skills in the absence of technology *(2 missing)*	30 (35.9)
Improved cultural competency *(2 missing)*	55 (72.4)
Improved public health understanding *(2 missing)*	54 (71.1)
Improved research skills *(3 missing)*	9 (12.0)
Improved language skills *(2 missing)*	31 (40.8)

## Discussion

In this analysis of survey and medical school application data, we found that participants who reported completing a global elective during medical school were 22% more likely to report currently working with underserved populations. Completion of a work or volunteer experience with global focus *before* entering medical school was not strongly associated with current work with underserved populations. Medical school graduates who participated in electives in global settings also reported several benefits of these experiences, including building cultural understanding and language skills, having a better understanding of public health, and improved clinical and diagnostic skills in the absence of technology.

This association between completing a global elective as medical students and later working with underserved populations remained statistically significant after adjusting for experiences prior to medical school. We performed a sensitivity analysis excluding those still in clinical training programs and found that practicing clinicians who completed global health electives as residents were highly likely to have done so in medical school as well. Given the temporal nature of the electives with respect to subsequent career choices, it is possible that medical school electives indeed spur an increased interest in global health careers. Our findings are consistent with prior studies that similarly found an association between a medical school global elective experience and an intention of working or living in an underserved setting [4, 11]. However, these studies were limited to the “intent” to practice rather than actual practice outcome. Our study surveyed medical school alumni 4 to 8 years after graduation and we ascertained actual practice outcome. Omoren, et al. surveyed physicians and found that as compared to controls, physicians who had performed a two-month elective in Kenya during medical school were more likely to pursue primary care careers with underserved populations (2015).

When asked about their perceptions of their global health experiences and their influence in getting the jobs they wanted, participants noted several benefits, including improved cultural competency  and clinical and diagnostic skills despite technological limitations. None of the participants reported the program had any negative impact on their career choices, though some participants reported that their experiences did not have any influence on their ability to get the job they wanted. The perceived benefits of global health experiences might differ based upon one’s sense of duty to serve communities in need, that is, whether the student was motivated through an interest in serving a particular community or not. More information is needed on how medical students define service to others and how they apply this throughout their career choices and ongoing work activities.

A recent study of the career intentions of over 40,000 medical school graduates found that underrepresented racial and ethnic groups were nearly twice as likely as white students to report working with underserved populations [2]. While race and ethnicity were not associated with the outcome in this study’s bivariate analysis, the small number of Asian, Black, Indigenous (*n* = 39), and Latinx (*n* = 6) participants did not lend us the capability to fully test this within our school. Future studies may need to oversample participants from Black, Asian and Indigenous and Latinx groups to better understand predictors of their engaging in work with underserved populations, and qualitative research in smaller samples can elucidate motivations for choosing this career path or not.

Four participants reported conducting global health practice in an international setting after completing a global elective in medical school, while most participants (*n* = 121) reported clinical practice, research, teaching, or programmatic work with a global focus within the U.S. Further research can supplement these data to better understand factors identifying global health engagement. This study also has implications for global health education in the medical school setting, including a better understanding of the barriers to global health careers and how we can help trainees surmount these barriers.

A limitation of this study is that it was conducted among a moderately-sized sample of graduates from a single medical school, and the findings might not be applicable to all medical school contexts. Future research should incorporate data from a variety of medical schools with variation by geography, public/private status, and applicant characteristics. Medical students interested in working with underserved populations might self-select into certain programs based on institutional factors, and our single-site study cannot necessarily capture those nuances. A small sample size led to imprecise prevalence ratio estimates that must be interpreted with caution. We attempted to maximize the response rate by extending the survey deadline and increasing the incentive amount; however, we only achieved a final response rate of 21.7%. A post-hoc comparison showed that respondents and non-respondents were comparable with respect to age, though women were more likely to respond than men (data not shown). In a survey of 161 graduates of this medical school graduating class of 2014, 5 years post-graduation, 67 (41.6%) continued to practice in-state. Of these, 5 were practicing in rural health settings and 4 were practicing in safety net settings that provide care to vulnerable populations; 9 worked in the most economically distressed settings in the state. These data suggest that work with underserved populations is generally rare among graduates of this medical school, and that our survey might have oversampled participants working in these settings. As most participants reported working with immigrant populations in the United States (three-quarters of these reporting doing so between 1 and 25% of their working hours) it is possible that alumni currently involved in work with underserved populations were more motivated to respond to the survey. We also can not discount the possibility that unpleasant global health experiences might have had a negative impact on medical students’ intentions to practice in underserved populations, which might also have affected our response rate. Alternative data collection methods may support the validity of our findings, such as analysis of existing data and qualitative research to gather in-depth data from a smaller number of participants.

## Conclusion

In a sample of graduates from a large, public medical school on the east coast, having completed a global health elective during medical school was associated with a 22% increase in pursuing a career working with underserved populations, while few students overall pursued full-time clinical careers in global settings. Participants who participated in these electives reported multiple benefits, namely improved cultural humility  and deepened  public health knowledge. Growing and strengthening global health opportunities for medical students will provide opportunities to assess the impact of these experiences in pursuing careers dedicated to underserved populations in the U.S. and abroad. Medical school administrators and educators can use these data to guide curriculum planning around global health electives and may want to focus learning objectives on strengthening skills that could be used in clinical practice both outside and inside the United States.

## Supplementary Information

**Additional file 1.** Global Health Student Alumni Questionnaire

## Data Availability

The study team obtained permission from our school of medicine’s administrators to access data that students provided in their American Medical College Application Services (AMCAS) applications when they had applied to medical schools. The link to the 2021 AMCAS application guide for 2021 can be accessed here: https://students-residents.aamc.org/media/5186/download.

## Abbreviation

AMCAS: American Medical College Application Services
