# Supplementary material for: Students with global experiences during medical school are more likely to work in settings that focus on the underserved: an observational study from a public U.S. institution
Source: BMC Med Educ. 2021 Oct 29;21:552. doi: 10.1186/s12909-021-02975-3 (PMC8556999; doi:10.1186/s12909-021-02975-3)
Supplement: Supplementary file 1 — Additional file 1. Global Health Student Alumni Questionnaire [file 12909_2021_2975_MOESM1_ESM.docx]

Start of Block: SURVEY INSTRUCTIONS

Q1 Medical Student Alumni Survey  
 Welcome to the School of Medicine's Global Health Alumni Survey. Upon completing, you will be receiving a $20 coffee E-gift card sent to your email.

 The purpose of this survey is to understand careers of School of Medicine Alumni who did and did not participate in global health programs during medical school.
  
 How will your responses be used & your data protected?  The information from this survey will be used by the Office of Global Health Education in accordance with Institutional Review Board to evaluate School of Medicine global programs by knowing something about the careers of alumni. Your participation in this survey is voluntary and your names will not be linked with any personal, identifying information. This survey is being administered to all alumni from 2011-2015. The SOM may use email addresses in confidentiality to help us better reach alumni with future questionnaires. 

 Timeline: This survey takes only about 9 minutes to complete. It will remain available online for ten weeks, closing July 23, 2019. Upon completion, participants will receive a $20 coffee E-gift card via email.

 *Should you experience technical difficulties, have other questions regarding the survey, or would like to receive the survey results, please contact School of Medicine's Office of Global Health Education: Shay_Slifko@med.unc.edu*

|  |
| --- |

Q2 Knowing the purpose of the survey, do you wish to participate?

- Yes, I agree to participate. (1)
- No, I do not wish to participate and would like to exit this survey. (0)

*Skip To: End of Survey If Knowing the purpose of the survey, do you wish to participate? = No, I do not wish to participate and would like to exit this survey.*

*Skip To: End of Block If Knowing the purpose of the survey, do you wish to participate? = Yes, I agree to participate.*

End of Block: SURVEY INSTRUCTIONS

Start of Block: 2. Current Practice

|  |
| --- |

Q3 Are you currently employed?

- Yes (1)
- No (0)

*Skip To: End of Block If Are you currently employed? = No*

| 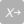 | 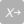 |
| --- | --- |

Q4 Select your current job position. Select all that apply.

- Resident (1)
- Fellow (2)
- Physician in outpatient setting (3)
- Physician in inpatient setting (4)
- Administrative leadership (5)
- Other- please specify (6) ________________________________________________

| 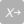 |
| --- |

Q5 Are you currently practicing in the United States?

- Yes (1)
- No. Where are you practicing? (0) ________________________________________________

*Display This Question:*

*If Are you currently practicing in the United States? = Yes*

| 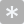 |
| --- |

Q6 Please enter the local zip code where you are practicing most often.

________________________________________________________________

*Display This Question:*

*If Are you currently practicing in the United States? = No. Where are you practicing?*

Q7 Please enter the city and country where you are practicing.

- City (4) ________________________________________________
- Country (5) ________________________________________________

| 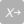 | 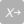 |
| --- | --- |

Q8 Are you working in a special type of practice?

- Federal Health Professional Shortage Area (HPSA) (1)
- State-designated shortage area (2)
- FEDERALLY DESIGNATED Veterans Administration facility Federally qualified Community Health Center (FQHC) Migrant Health Center (MHC) Indian Health Services (IHS) Clinic or Hospital Rural Health Clinic Critical Access Hospital (3)
- NOT FEDERALLY DESIGNATED Academic practice Community Health Center (NOT federally designated) Tribal Clinic; urban Indian clinic Correctional facility Health Department Community Mental health facility Other (4)
- No. (5)

*Display This Question:*

*If Are you working in a special type of practice? = FEDERALLY DESIGNATED Veterans Administration facility Federally qualified Community Health Center (FQHC) Migrant Health Center (MHC) Indian Health Services (<strong>IHS</strong>) Clinic or Hospital Rural Health Clinic Critical Access Hospital*

Q9 Please provide Federal Designation below:   Select all that apply.

- Veterans Administration medical center (1)
- Federally qualified Community Health Center (FQHC) (2)
- Migrant Health Center (MHC) (3)
- Indian Health Services (IHS) Clinic or Hospital (4)
- Rural Health Clinic (5)
- Critical Access Hospital (6)

*Display This Question:*

*If Are you working in a special type of practice? = NOT FEDERALLY DESIGNATED Academic practice Community Health Center (NOT federally designated) Tribal Clinic; urban Indian clinic Correctional facility Health Department Community Mental health facility Other*

Q10 Please provide Non-Federal Designation below:  Select all that apply.

- Academic practice (1)
- Community Health Center (NOT federally designated) (2)
- Tribal Clinic; urban Indian clinic (3)
- Correctional facility (4)
- Health Department (5)
- Community Mental health facility (6)
- Other (7)

*Display This Question:*

*If Please provide Non-Federal Designation below:  Select all that apply. = Other*

Q11 Other:

________________________________________________________________

| 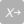 |
| --- |

Q12 Please indicate which medical specialty best describes your residency/ fellowship training program.
 (Hold CTRL to select multiple items)

- Anesthesiology or subspecialty (1)
- Dermatology or subspecialty (2)
- Emergency Medicine or subspecialty (3)
- Family Medicine - general (4)
- Family Medicine - subspecialty (5)
- Internal Medicine - general (6)
- Internal Medicine sub-specialty (7)
- Internal Medicine/Pediatrics (8)
- Medical Genetics or subspecialty (9)
- Neurological Surgery (10)
- Neurology or subspecialty (11)
- Nuclear Medicine (12)
- Obstetrics and Gynecology or subspecialty (13)
- Ophthalmology or subspecialty (14)
- Orthopedic Surgery or subspecialty (15)
- Otolaryngology or subspecialty (16)
- Pathology or subspecialty (17)
- Pediatrics - general (18)
- Pediatrics subspecialty (19)
- Physical Medicine or Rehabilitation or subspecialty (20)
- Plastic Surgery or subspecialty (21)
- Preventive Medicine or subspecialty (22)
- Psychiatry or subspecialty (23)
- Radiation Oncology (24)
- Radiology or subspecialty (25)
- Surgery or subspecialty (26)
- Thoracic Surgery or subspecialty (27)
- Urology or subspecialty (28)
- Vascular Surgery (29)
- Specialty not yet known (0)
- Not certain I will undertake residency (30)

| 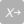 | 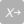 |
| --- | --- |

Q13  Please estimate the percentage of your work activities per year that are regularly dedicated to global health scholarly/academic/clinical, work, etc.?

|  | None (0) | 1-25% (1) | 26-50% (2) | 51-75% (3) | 76-100% (4) |
| --- | --- | --- | --- | --- | --- |
| Clinical activities conducted internationally (2) |  |  |  |  |  |
| Global health academic teaching (4) |  |  |  |  |  |
| Global health research conducted from the U.S. (1) |  |  |  |  |  |
| Global health programmatic work with NGOs or other organizations (11) |  |  |  |  |  |
| Working with immigrant populations in the U.S. (8) |  |  |  |  |  |
| Other (7) |  |  |  |  |  |

Q14 Approximately, what percentage of your patient population is insured under: *(Please make your best estimate)*

- Medicaid % (2) ________________________________________________
- Medicare % (14) ________________________________________________
- Champ us or Tricare (military) coverage % (15) ________________________________________________
- Private (non-public) health insurance (16) ________________________________________________
- Indian Health Service or tribal coverage % (17) ________________________________________________
- Uninsured % (10) ________________________________________________
- Other type of coverage % (3) ________________________________________________
- N/A- I do not see patients. (13) ________________________________________________

Q15 Approximately, what percentage of your patient population require an English-language translator or language-proficient provider?

- Enter percentage (12) ________________________________________________

| Page Break |  |
| --- | --- |

| 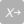 |
| --- |

Q16 Have you developed or produced any resources (books, publications, articles, courses, websites, etc.) focused on your international area of expertise?

- Yes (1)
- No (0)

*Display This Question:*

*If Have you developed or produced any resources (books, publications, articles, courses, websites, e... = Yes*

Q17 Please describe up to 10 resources you developed since medical school graduation and include any web links. Resources may include articles, book chapters, courses, or websites.

________________________________________________________________

________________________________________________________________

________________________________________________________________

________________________________________________________________

________________________________________________________________

End of Block: 2. Current Practice

Start of Block: 3. Did a global elective inform your current practice?

| 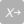 |
| --- |

Q18 During medical school, did you participate in a global health elective or global program abroad?  

- Yes (1)
- No (0)

*Skip To: Q30 If During medical school, did you participate in a global health elective or global program abroad? ... = No*

| 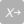 | 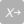 |
| --- | --- |

Q19 What was the total length of time you were at an international site in a medical student role?

- < 1 week (0)
- 1-2 weeks (1)
- 1 month (2)
- 2 months (3)
- 3 months (4)
- 3-6 months (5)
- 6 months to 1 year (6)

| 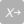 |
| --- |

Q20 What was your role during this international experience as a medical student? Select all that apply.

- Clinical work in a hospital (1)
- Clinical work in an outpatient setting (2)
- Research (3)
- Improving language skills (4)
- Public health or community health project (5)
- Other- Please specify (6) ________________________________________________

| 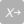 |
| --- |

Q21
How important has your medical school global experience been to your work since residency?

- Not at all important (1)
- Slightly important (2)
- Moderately important (3)
- Very important (4)
- Extremely important (5)

Q22 What was the biggest challenge encountered once you were on site?

________________________________________________________________

________________________________________________________________

________________________________________________________________

________________________________________________________________

________________________________________________________________

| 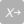 |
| --- |

Q23 Please indicate how important each reason below was to your participation in your global health experience(s) during medical school.  (We want to know YOUR reasons.)

|  | Not at all important (1) | Slightly important (2) | Important (3) | Moderately Important (4) | Very important (5) |
| --- | --- | --- | --- | --- | --- |
| Research experience (1) |  |  |  |  |  |
| Develop clinical skills (2) |  |  |  |  |  |
| Interest in travel to that region (3) |  |  |  |  |  |
| Developing skills in another language (4) |  |  |  |  |  |
| Family/ethnic background in that region (5) |  |  |  |  |  |
| Faith/Religion (6) |  |  |  |  |  |
| Build CV for residency (7) |  |  |  |  |  |
| Sense of service (9) |  |  |  |  |  |
| Other (8) |  |  |  |  |  |

| 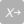 |
| --- |

Q24 How important was your medical school global experience in choosing your residency?

- Not at all important (1)
- Slightly important (2)
- Moderately important (3)
- Very important (4)
- Extremely important (5)

| 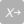 | 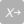 |
| --- | --- |

Q25
Select the answer most closely associated with your personal experiences. Select a single response for each line.
 
My global health elective experience(s)...

|  | No (1) | Slightly (2) | Moderately (3) | Substantially (4) | Yes, profoundly (5) |
| --- | --- | --- | --- | --- | --- |
| Improved my clinical skills. (Q25_1) |  |  |  |  |  |
| Helped my diagnostic skills in the absence of technology. (Q25_2) |  |  |  |  |  |
| Improved cultural competency skills. (Q25_3) |  |  |  |  |  |
| Improved my understanding of public health. (Q25_4) |  |  |  |  |  |
| Improved my research skills. (Q25_5) |  |  |  |  |  |
| Improved my language skills. (Q25_6) |  |  |  |  |  |

Q26 Please state if and how your global health experience(s) during medical school have affected (positively or negatively) your ability to get a job you wanted?

________________________________________________________________

________________________________________________________________

________________________________________________________________

________________________________________________________________

________________________________________________________________

| Page Break |  |
| --- | --- |

Q27 Please provide one or more examples of how your global experience(s) at your medical school improved your clinical care of patients.

________________________________________________________________

________________________________________________________________

________________________________________________________________

________________________________________________________________

________________________________________________________________

Q28 In what region did you participate in a global elective?
 (Hold CTRL to select multiple items)

- Africa (1)
- Central Asia (2)
- East Asia (3)
- Latin America/Caribbean (4)
- Middle East (5)
- Oceania (6)
- Russia, Eastern Europe or Eurasia (7)
- South Asia (8)
- Southeast Asia (9)
- Western Europe (10)
- N/A- Domestic Only (11)

| 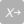 |
| --- |

Q29 How often do you use your region-specific knowledge in your current role?

- Almost never (1)
- Less than once a month (2)
- Once a month (3)
- Several times a month (4)
- Several times a week (5)

*Display This Question:*

*If During medical school, did you participate in a global health elective or global program abroad? ... = No*

| 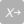 |
| --- |

Q30 Why didn't you or couldn't you pursue a global program abroad during medical school. Select all that apply.

- I had no interest in a global experience (1)
- Didn't know about opportunities (2)
- Started the process but stopped because it was burdensome (3)
- Applied for programs but wasn't accepted (4)
- Worried that I wouldn't complete my medical school on time (5)
- Did not have the funds to participate (6)
- Faced the burden of lost working wages (7)
- Lack of support, encouragement, or understanding from family members (8)
- Decided there was little value of global travel for my medical education (9)
- Lack of peer support (10)
- Lack of faculty support (11)
- Concern about this negatively impacting my chances to match in competitive residency programs (12)
- Concern about safety at international location (13)
- My academic standing was unclear and therefore was not ideal for me (14)
- Other, please specify (15) ________________________________________________

*Display This Question:*

*If During medical school, did you participate in a global health elective or global program abroad? ... = No*

Q31 Please share more about why you did not have an interest in a global experience.

________________________________________________________________

________________________________________________________________

________________________________________________________________

________________________________________________________________

________________________________________________________________

| 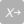 | 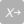 |
| --- | --- |

Q32 Languages you speak fluently:
(Hold CTRL to select multiple items)

- Arabic (1)
- Bosnian-Croatian-Serbian (2)
- Chinese (3)
- French (4)
- German (5)
- Greek, Modern (6)
- Haitian Creole (7)
- Hebrew, Modern (8)
- Hindi (9)
- Italian (10)
- Japanese (11)
- Kiswahili (12)
- Korean (13)
- Lingala (14)
- Persian (15)
- Polish (16)
- Portuguese (Brazilian) (17)
- Portuguese (Iberian) (18)
- Russian (19)
- Spanish (20)
- Tagalog (21)
- Turkish (22)
- Urdu (23)
- Vietnamese (24)
- Wolof (25)
- Yucatec Maya (26)
- Other (Please indicate language(s) spoken fluently) (28) ________________________________________________

| 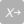 | 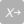 |
| --- | --- |

Q33 In your current role in clinical care, administrative, and all other work role, how often do you use the language(s)? Select ONLY those that apply.

|  | Less than once a month (1) | Once a month (2) | Weekly (3) | 2/3 times a week (4) | Daily (5) |
| --- | --- | --- | --- | --- | --- |
| Arabic (Q34_1) |  |  |  |  |  |
| Bosnian-Croatian-Serbian (Q34_43) |  |  |  |  |  |
| Chinese (Q34_6) |  |  |  |  |  |
| French (Q34_10) |  |  |  |  |  |
| German (Q34_11) |  |  |  |  |  |
| Greek, modern (Q34_44) |  |  |  |  |  |
| Haitian Creole (Q34_33) |  |  |  |  |  |
| Hebrew, modern (Q34_45) |  |  |  |  |  |
| Hindi (Q34_17) |  |  |  |  |  |
| Italian (Q34_19) |  |  |  |  |  |
| Japanese (Q34_46) |  |  |  |  |  |
| Kiswahili (Q34_47) |  |  |  |  |  |
| Korean (Q34_22) |  |  |  |  |  |
| Lingala (Q34_48) |  |  |  |  |  |
| Polish (Q34_42) |  |  |  |  |  |
| Portuguese (Brazilian) (Q34_29) |  |  |  |  |  |
| Portuguese (Iberian) (Q34_30) |  |  |  |  |  |
| Russian (Q34_31) |  |  |  |  |  |
| Tagalog (Q34_35) |  |  |  |  |  |
| Turkish (Q34_49) |  |  |  |  |  |
| Urdu (Q34_36) |  |  |  |  |  |
| Vietnamese (Q34_38) |  |  |  |  |  |
| Wolof (Q34_50) |  |  |  |  |  |
| Yucatec Mayan (Q34_51) |  |  |  |  |  |
| Other: Please indicate (Q34_39) |  |  |  |  |  |
| Spanish (Q34_52) |  |  |  |  |  |

| 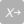 |
| --- |

Q34 Regarding patient population, have you spent at least 5% of your time (in work or volunteer situations) with any of the following populations since completing medical school?

|  | Yes (1) | No (0) |
| --- | --- | --- |
| Medically under­served (1) |  |  |
| Immigrants (2) |  |  |
| Non-English speakers (3) |  |  |
| Ethnic or racial minorities (4) |  |  |
| Prison inmates (5) |  |  |
| Homeless population (6) |  |  |

End of Block: 3. Did a global elective inform your current practice?

Start of Block: 4. Future Global Engagement

| 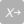 |
| --- |

Q35 During residency, did you do a global health elective?

- Yes. Please share where and what type of rotation? (1) ________________________________________________
- No (0)

*Skip To: Q37 If During residency, did you do a global health elective? = Yes. Please share where and what type of rotation?*

*Display This Question:*

*If During residency, did you do a global health elective? = No*

| 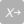 |
| --- |

Q36
What deterred you from pursuing a global health elective during residency? Select all that apply.

- I'm still in residency training and will NOT pursue global health activities (1)
- I'm still in residency training and may pursue global health activities (2)
- No resources for global health efforts at current job/training (3)
- No interest in global health engagement (4)
- Too many loans (5)
- Concerned about losing clinical privileges (6)
- No extra time (7)
- No one to cover my patient panel while away (8)
- Did not want to lose salary (9)
- Concern about safety at international location (10)
- Partner and/or children could not travel with me (11)
- Other, please specify (12) ________________________________________________

| 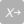 | 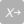 |
| --- | --- |

Q37 What was the (approximate) total of your student loan debt at the time of your medical school graduation?

- None (1)
- Less than $25,000 (2)
- $25,000-$49,999 (3)
- $50,000-$99,999 (4)
- $100,000-$199,000 (5)
- $200,000-299.999 (6)
- $300,000-399,999 (7)
- $400,000 or more (8)

End of Block: 4. Future Global Engagement

Start of Block: 5. School of Medicine global health support

| 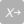 |
| --- |

Q38 Did you interact with the School of Medicine's Office of Global Health Education while planning your international experience?

- Yes (1)
- No (0)

*Skip To: Q41 If Did you interact with the School of Medicine's Office of Global Health Education while planning your global experience?*

- Yes (1)
- No (0)

| 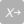 |
| --- |

| 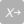 | 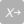 |
| --- | --- |

Q40 How would you rate your satisfaction with the medical school’s Office of Global Health Education in each of the following ways?

|  | Very Dissatisfied (1) | Moderately Dissatisfied (2) | Satisfied (4) | Moderately Satisfied (5) | Very Satisfied (6) |
| --- | --- | --- | --- | --- | --- |
| Clarity in explaining the global health elective process (Q42_1) |  |  |  |  |  |
| Timely Communication (Q42_2) |  |  |  |  |  |
| Professionalism (Q42_3) |  |  |  |  |  |

Q41 How could the SOM Office of Global Health Education have better supported you during medical school?

________________________________________________________________

________________________________________________________________

________________________________________________________________

________________________________________________________________

________________________________________________________________

End of Block: 5. School of Medicine global health support

Start of Block: Background Information

Q42 What was your name when you were a student?

- First Name (1) ________________________________________________
- Last Name (2) ________________________________________________

| 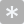 |
| --- |

Q43 What is your preferred email address?

________________________________________________________________

| 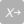 |
| --- |

Q44 In which year did you receive your medical degree?

▼ 2016 (10) ... 2011 (5)

| 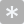 |
| --- |

Q45 What year were you born? *(Use format: mm/dd/yyyy)*

________________________________________________________________

| 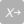 |
| --- |

Q46
Please check if you have completed any of the following formal training relevant to global health. Select all that apply.

- MPH degree (0)
- Another masters or doctorate directly relevant to a global health degree (1)
- A global health certificate program (2)

| 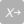 |
| --- |

Q47 How many people are included in your household (include yourself in the count).

▼ 1 (1) ... Prefer not to answer (99)

| 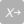 |
| --- |

Q48 Please indicate your race. Select all that apply.

- Alaska Native (1)
- American Indian or American Indian (2)
- Asian (3)
- Black or African American (4)
- Native Hawaiian or other Pacific Islander (5)
- White or Caucasian (6)
- Not listed above (7) ________________________________________________
- Prefer not to answer (99)

| 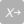 |
| --- |

Q49 Are you of Hispanic or Latino(a) ethnicity?

- Yes (1)
- No (0)
- Prefer not to answer (99)

| 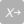 |
| --- |

Q50  Please indicate your partnership/relationship status.

- Divorced (1)
- Domestic partnership/ co-habitating (2)
- Married/civil union (3)
- Separated (4)
- Single/not married (5)
- Widowed (6)
- Not listed above (7) ________________________________________________
- Prefer not to answer (99)

| 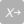 |
| --- |

Q51 Please indicate your gender identity.

- Female (1)
- Male (2)
- Nonbinary (3)
- Not listed here: (4) ________________________________________________
- Prefer not to answer (99)

Q52 This concludes the survey. Please click the Submit button and you will be automatically entered to receive a $10 gift card. Thanks!

End of Block: Background Information
